# Prospective cohort study of referred Malawian children and their survival by hypoxaemia and hypoglycaemia status

**DOI:** 10.2471/BLT.21.287265

**Published:** 2022-03-25

**Authors:** Carina King, Beatiwel Zadutsa, Lumbani Banda, Everlisto Phiri, Eric D McCollum, Josephine Langton, Nicola Desmond, Shamim Ahmad Qazi, Yasir Bin Nisar, Charles Makwenda, Helena Hildenwall

**Affiliations:** a Department of Global Public Health, Karolinska Institutet, Tomtebogatan 18a, Stockholm, 17177, Sweden.; b Parent and Child Health Initiative, Lilongwe, Malawi.; c Global Program in Respiratory Sciences, Johns Hopkins University, Baltimore, United States of America.; d Department of Paediatrics, College of Medicine, Blantyre, Malawi.; e Behaviour and Health Group, Malawi-Liverpool-Wellcome Trust Programme, Blantyre, Malawi.; f Department of Maternal, Newborn, Child and Adolescent Health and Ageing, World Health Organization, Geneva, Switzerland.

## Abstract

**Objective:**

To investigate survival in children referred from primary care in Malawi, with a focus on hypoglycaemia and hypoxaemia progression.

**Methods:**

The study involved a prospective cohort of children aged 12 years or under referred from primary health-care facilities in Mchinji district, Malawi in 2019 and 2020. Peripheral blood oxygen saturation (SpO_2_) and blood glucose were measured at recruitment and on arrival at a subsequent health-care facility (i.e. four hospitals and 14 primary health-care facilities). Children were followed up 2 weeks after discharge or their last clinical visit. The primary study outcome was the case fatality ratio at 2 weeks. Associations between SpO_2_ and blood glucose levels and death were evaluated using Cox proportional hazards models and the treatment effect of hospitalization was assessed using propensity score matching.

**Findings:**

Of 826 children recruited, 784 (94.9%) completed follow-up. At presentation, hypoxaemia was moderate (SpO_2_: 90–93%) in 13.1% (108/826) and severe (SpO_2_: < 90%) in 8.6% (71/826) and hypoglycaemia was moderate (blood glucose: 2.5–4.0 mmol/L) in 9.0% (74/826) and severe (blood glucose: < 2.5 mmol/L) in 2.3% (19/826). The case fatality ratio was 3.7% (29/784) overall but 26.3% (5/19) in severely hypoglycaemic children and 12.7% (9/71) in severely hypoxaemic children. Neither moderate hypoglycaemia nor moderate hypoxaemia was associated with mortality.

**Conclusion:**

Presumptive pre-referral glucose treatment and better management of hypoglycaemia could reduce the high case fatality ratio observed in children with severe hypoglycaemia. The morbidity and mortality burden of severe hypoxaemia was high; ways of improving hypoxaemia identification and management are needed.

## Résumé

**Objectif:**

Analyser le taux de survie des enfants transférés depuis les soins primaires au Malawi, en se focalisant sur l'évolution de l'hypoglycémie et de l'hypoxémie.

**Méthodes:**

Cette étude a été menée sur une cohorte prospective d'enfants âgés de maximum 12 ans et ayant fait l'objet d'un transfert depuis des établissements de soins primaires situés dans le district de Mchinji, au Malawi, en 2019 et 2020. La saturation en oxygène du sang périphérique (SpO_2_) et la glycémie ont été mesurées lors de la sélection ainsi qu'à l'arrivée dans le centre de soins suivant (c'est-à-dire l'un des quatre hôpitaux ou des 14 établissements de soins de santé primaires). Les enfants étaient suivis deux semaines après leur sortie ou leur dernière visite clinique. Le résultat de l'étude primaire correspond au taux de létalité à deux semaines. Nous avons utilisé des modèles à risques proportionnels de Cox pour identifier les liens entre la SpO_2_, le taux de glycémie et le décès, et un appariement des coefficients de propension pour évaluer l'effet thérapeutique de l'hospitalisation.

**Résultats:**

Sur 826 enfants sélectionnés, 784 (94,9%) sont arrivés au terme du suivi. Au moment de leur présentation, l'hypoxémie était modérée (SpO_2_: 90–93%) chez 13,1% (108/826) et sévère (SpO_2_: < 90%) chez 8,6% d'entre eux (71/826). De son côté, l'hypoglycémie était modérée (glycémie: 2,5–4,0 mmol/L) chez 9,0% (74/826) et sévère (glycémie: < 2,5 mmol/L) chez 2,3% d'entre eux (19/826). Le taux de létalité s'élevait à 3,7% (29/784) au total, mais à 26,3% (5/19) chez les enfants souffrant d'hypoglycémie sévère et à 12,7% (9/71) chez ceux souffrant d'hypoxémie sévère. Ni l'hypoglycémie modérée, ni l'hypoxémie modérée n'étaient associées au décès.

**Conclusion:**

Un traitement présomptif avant le transfert et une meilleure gestion de l'hypoglycémie pourraient réduire le haut taux de létalité observé chez les enfants souffrant d'hypoglycémie sévère. La charge de morbidité et de mortalité imputable à l'hypoxémie sévère était considérable; il est donc impératif d'améliorer le diagnostic et la gestion de l'hypoxémie.

## Resumen

**Objetivo:**

Estudiar la supervivencia de los niños remitidos desde la atención primaria en Malawi, con un enfoque en la evolución de la hipoglucemia y la hipoxemia.

**Métodos:**

El estudio incluyó una cohorte prospectiva de niños de 12 años o menos remitidos desde centros de atención primaria de salud ubicados en el distrito de Mchinji, Malawi, en 2019 y 2020. La saturación de oxígeno en sangre periférica (SpO_2_) y la glucemia se midieron en el momento de selección y a la llegada a un centro sanitario posterior (es decir, cuatro hospitales y 14 centros de atención primaria). Se realizó un seguimiento de los niños dos semanas después del alta o de su última visita médica. El resultado principal del estudio fue la tasa de mortalidad a las dos semanas. Las asociaciones entre los niveles de SpO_2_ y de glucemia y la muerte se evaluaron mediante modelos de riesgo proporcional de Cox, y el efecto del tratamiento de la hospitalización se evaluó mediante el emparejamiento del índice de propensión.

**Resultados:**

De los 826 niños seleccionados, 784 (94,9 %) completaron el seguimiento. Cuando se presentaron, la hipoxemia era moderada (SpO_2_: 90-93 %) en el 13,1 % (108/826) y grave (SpO_2_: <90 %) en el 8,6 % (71/826), mientras que la hipoglucemia era moderada (glucemia: 2,5-4,0 mmol/l) en el 9,0 % (74/826) y grave (glucemia: <2,5 mmol/l) en el 2,3 % (19/826). La tasa de letalidad fue del 3,7 % (29/784) en general, pero del 26,3 % (5/19) en los niños con hipoglucemia grave y del 12,7 % (9/71) en los niños con hipoxemia grave. Ni la hipoglucemia moderada ni la hipoxemia moderada se asociaron a la mortalidad.

**Conclusión:**

El tratamiento presunto de la glucosa antes de la remisión y el mejor tratamiento de la hipoglucemia podrían reducir la elevada tasa de letalidad que se observa en los niños con hipoglucemia grave. La carga de morbilidad y mortalidad de la hipoxemia grave fue elevada; en consecuencia, es preciso mejorar la identificación y el tratamiento de la hipoxemia.

## ملخص

**الغرض:**

للاستقصاء في شأن بقاء الأطفال المُحالين من الرعاية الأولية في ملاوي على قيد الحياة، مع التركيز على تطور حالة نقص السكر ونقص تأكسج الدم.

**الطريقة:**

شملت الدراسة مجموعة إترابية من الأطفال في سن 12 عامًا أو أقل، من المُحالين من مرافق الرعاية الصحية الأولية في منطقة Mchinji، في ملاوي في عامي 2019 و2020. تم قياس كل من تشبع الأكسجين في الدم المحيطي (SpO _2 _)، ونسبة الجلوكوز في الدم عند التجنيد وعند الوصول إلى مرفق رعاية صحية لاحق (أي أربعة مستشفيات، و14 مرفقاً للرعاية الصحية الأولية). تمت متابعة الأطفال بعد أسبوعين من الخروج، أو بعد آخر زيارة سريرية لهم. كانت نتيجة الدراسة الأولية هي نسبة الوفيات للحالات بعد أسبوعين. تم تقييم الارتباطات بين SpO _2 _ ومستويات الجلوكوز في الدم والوفاة، باستخدام نماذج المخاطر النسبية Cox، وتم تقييم تأثير العلاج في المستشفى باستخدام مطابقة درجة الميل.

**النتائج:**

من بين 826 طفلاً تم إشراكهم، أكمل 784 (94.9%) منهم المتابعة. عند التقديم، كان نقص تأكسج الدم معتدلاً (SpO _2 _: 90 إلى 93%) في 13.1% (108/826) وشديدًا (SpO _2 _: أقل من 90%) في 8.6% (71/826) وكان نقص سكر الدم معتدلاً (جلوكوز الدم: 2.5 إلى 4.0 مليمول/لتر) في 9.0% (74/826) وحاد (جلوكوز الدم: أقل من 2.5 مليمول/لتر) في 2.3% (19/826). كانت نسبة الوفيات للحالات 3.7% (29/784) بشكل عام، ولكن 26.3% (5/19) في الأطفال المصابين بنقص شديد في سكر الدم، و12.7% (9/71) في الأطفال المصابين بنقص شديد في تأكسج الدم. لم يرتبط نقص سكر الدم المعتدل، أو نقص تأكسج الدم المعتدل، بالوفيات.

**الاستنتاج:**

يمكن للمعالجة الافتراضية للجلوكوز قبل الإحالة، والتحكم الأفضل في نقص سكر الدم، أن يقللا من نسبة وفيات الحالات المرتفعة التي لوحظت في الأطفال المصابين بنقص شديد في سكر الدم. أدى نقص تأكسج الدم الحاد إلى عبء مرتفع للإصابة بالأمراض والوفيات؛ حيث كانت هناك حاجة إلى طرق لتحسين التعرف على نقص تأكسج الدم ومعالجته.

## 摘要

**目的:**

调查马拉维从初级护理转诊的儿童的生存状况，重点关注其低血糖和低氧血的进展。

**方法:**

研究涉及 2019 年和 2020 年马拉维从姆钦吉区初级护理机构转诊的 12 岁及以下儿童的前瞻性群组。在招募时和随后到达卫生保健机构（即四家医院和 14 家初级卫生保健机构）时测量外周血氧饱和度 (SpO_2_) 和血糖浓度。在出院或最后一次就诊 2 周后对儿童进行随访。主要研究结果是 2 周后的病死率。使用 Cox 比例风险模型评估 SpO_2_ 和血糖水平与致死率之间的关联，使用倾向评分匹配方法评估住院治疗效果。

**结果:**

招募的 826 名儿童中，有 784 (94.9%) 名儿童完成了随访。就诊时，中度低氧血 (SpO_2_: 90–93%) 人数比例为 13.1% (108/826)，重度低氧血 (SpO_2_: < 90%) 为 8.6% (71/826)，中度低血糖（血糖浓度：2.5–4.0 毫克分子/升）为 9.0% (74/826)，重度低血糖（血糖浓度：< 2.5 mmol/L）为 2.3% (19/826)。总体病死率为 3.7% (29/784)，其中重度低血糖儿童比例为 26.3% (5/19)，重度低氧血儿童比例为 12.7% (9/71)。中度低血糖和中度低氧血均与死亡率的变化无关。

**结论:**

假定转诊前对儿童进行葡萄糖治疗和更好的低血糖诊治，可以降低重度低血糖儿童的高死亡率。重度低氧血的发病率和死亡率负担很高，需要改善低氧血症确诊和诊治的方法。

## Резюме

**Цель:**

Изучить выживаемость детей, направленных из учреждений первичной медико-санитарной помощи в Малави, с упором на гипогликемию и прогрессирование гипоксемии.

**Методы:**

В исследование была включена проспективная когорта детей в возрасте до 12 лет включительно, направленных из учреждений первичной медико-санитарной помощи в районе Мчинджи, Малави, в 2019 и 2020 годах. Насыщение кислородом периферической крови (SpO_2_) и уровень глюкозы в крови измерялись во время набора в исследование и по прибытии в соответствующее медицинское учреждение (четыре больницы и 14 учреждений первичной медико-санитарной помощи). Последующее наблюдение проводили через 2 недели после выписки или последнего визита ребенка в клинику. Первичным результатом исследования был показатель летальности через 2 недели. Взаимосвязь между SpO_2_, уровнями глюкозы в крови и смертностью оценивалась с использованием модели пропорциональных рисков Кокса. Терапевтический эффект госпитализации оценивался с использованием псевдорандомизации.

**Результаты:**

Из 826 детей, включенных в исследование, 784 (94,9%) прошли последующее наблюдение. На момент первичного осмотра гипоксемия была умеренной степени (SpO_2_: 90–93%) у 13,1% детей (108/826) и тяжелой степени (SpO_2_: < 90%) у 8,6% детей (71/826). Гипогликемия была умеренной степени (глюкоза в крови: 2,5–4,0 ммоль/л) у 9,0% детей (74/826) и тяжелой степени (глюкоза в крови: < 2,5 ммоль/л) у 2,3% детей (19/826). Показатель летальности составил 3,7% (29/784) в целом, но 26,3% (5/19) у детей с тяжелой гипогликемией и 12,7% (9/71) у детей с тяжелой гипоксемией. Ни умеренная гипогликемия, ни умеренная гипоксемия не были связаны со смертностью.

**Вывод:**

Пробное догоспитальное лечение глюкозой и более эффективное лечение гипогликемии могут снизить высокий показатель летальности, наблюдаемый у детей с тяжелой гипогликемией. Бремя заболеваемости и смертности от тяжелой гипоксемии было высоким, поэтому необходимы способы совершенствования механизмов выявления и лечения гипоксемии.

## Introduction

Global initiatives to reduce child mortality have generally focused on improving early access to basic treatment for common illnesses using tools such as the World Health Organization’s (WHO) Integrated Management of Childhood Illness strategy and integrated community case management.^1^^–^^3^ In the absence of gold-standard diagnostic techniques for conditions such as pneumonia, these approaches rely primarily on subjective clinical assessment for syndromic case management. Children with signs of severe illness are referred to hospital for supportive care. However, emergency care is weak in under-resourced health systems, referrals can be difficult for caregivers, there may be delays due to a lack of transportation, and financial barriers are common.^4^^–^^6^ Children may, therefore, arrive at referral hospitals when their illness is at a late stage and treatment may be less effective. In Malawi, 33% (39/118) of deaths reported among children at a tertiary referral hospital in 2017 occurred in the first 24 hours after admission.^7^

Hypoxaemia and hypoglycaemia are objective measures associated with paediatric mortality.^7–10^ Indeed, Integrated Management of Childhood Illness protocols include hypoxaemia, defined as peripheral blood oxygen saturation (SpO_2_) below 90%, as a referral criterion and the threshold for initiating oxygen treatment. General danger signs (Box 1) are inadequate for identifying hypoxaemia and children may not receive the oxygen they need.^12,13^ An SpO_2_ level below 93% has been associated with deaths in children with clinical pneumonia in sub-Saharan Africa but evidence is lacking on the optimal referral threshold.^10,14,15^

Similarly, Integrated Management of Childhood Illness protocols recognize the risk posed by hypoglycaemia to children and recommend presumptive pre-referral treatment for those with danger signs. Currently, WHO defines hypoglycaemia in a well-nourished child as a blood glucose concentration below 2.5 mmol/L^1^ though increased mortality has been reported in children admitted with higher concentrations.^8,9,16,17^ A recent trial in Malawi found that hypoglycaemia treatment in hospitalized children with a blood glucose concentration between 2.5 and 5.0 mmol/L was not associated with survival.^18^ More evidence is needed on how best to detect, monitor and treat hypoglycaemia in children.

Early detection of moderate hypoxaemia (SpO_2_ between 90 and 93%) and moderate hypoglycaemia (blood glucose concentration between 2.5 and 4.0 mmol/L) in primary care may help reduce child mortality. The aim of our study was to investigate the survival of children referred from primary health-care facilities in Malawi, with a focus on clinical progression in those who presented with moderate hypoglycaemia or moderate hypoxaemia.

## Methods

**Fig. 1:**
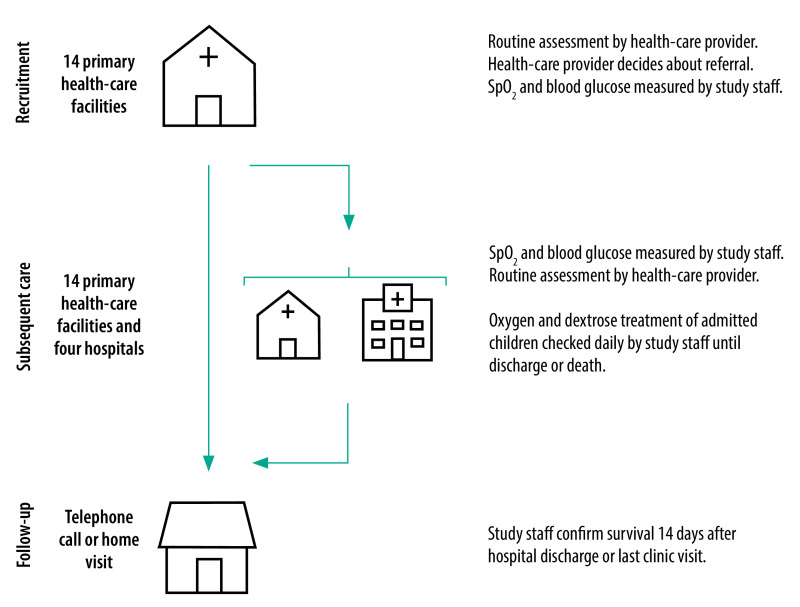
Recruitment and follow-up procedures, Malawi, 2019–2020

In this preplanned secondary analysis of a prospective cohort study, we assessed the survival of children referred from primary health-care facilities to hospitals in Mchinji district, Malawi. Mchinji had a population of approximately 600 000 in 2015 to 2016 and a mortality rate in children younger than 5 years of 123 per 1000 live births.^19^ In particular, we followed children with severe or moderate hypoxaemia or hypoglycaemia (Box 1) from recruitment to presentation at another facility (Fig. 1). Children were recruited at all 14 functional, government, primary health-care facilities in Mchinji district that provided outpatient paediatric care: two dispensaries, 11 health centres and one rural hospital with no inpatient care. Three rural hospitals and a district hospital acted as referral facilities. The nearest tertiary referral hospital was in the neighbouring district of Lilongwe (no data were collected from this facility). To be eligible for inclusion in the study, referred children had to be aged between 0 months and 12 years and be resident in Mchinji district. Recruitment started on 1 July 2019 and was intended to last 12 months. However, enrolment was terminated early, on 6 April 2020, because of the coronavirus disease 2019 (COVID-19) pandemic. Follow-ups were completed on 13 June 2020.

Box 1 **Terminology used in the prospective cohort study of survival in children with hypoxaemia and/or hypoglycaemia on referral, Malawi, 2019–2020**

**Study outcome**

Death: Death of a child from any cause between study recruitment and 14 days after hospital discharge or their last documented clinical visit, as recorded during hospital admission or in a follow-up interview

**Exposure**

Hypoxaemia

- Normoxaemia: SpO_2_: 94–100%
- Moderate hypoxaemia:^a^ SpO_2_: 90–93%
- Severe hypoxaemia: SpO_2_: < 90%^1^ (values < 50% were considered invalid)

Hypoglycaemia

- Normoglycaemia: Blood glucose concentration > 4.0 mmol/L
- Moderate hypoglycaemia:^a^ Blood glucose concentration 2.5–4.0 mmol/L in well-nourished and moderately malnourished children and 3.0–4.0 mmol/L in severely malnourished children
- Severe hypoglycaemia: Blood glucose concentration < 2.5 mmol/L in well-nourished and moderately malnourished children and < 3.0 mmol/L in severely malnourished children

**Other**

Danger signs^1^

- Child aged < 2 months:^b^ Any documentation in the child’s health passport or the caregiver’s report of the following signs: (i) inability to drink or feed; (ii) convulsions; (iii) movement only when stimulated or no movement at all; (iv) fast breathing (i.e. ≥  60 breaths per minute); (v) severe chest indrawing; and (vi) axillary temperature < 35.5 °C or *≥* 37.5 °C
- Child aged 2 months to 12 years:^c^ Any documentation in the child’s health passport or the caregiver’s report of the following signs: (i) vomiting everything; (ii) inability to drink or feed; (iii) convulsions; (iv) sleepy or lethargic; and (v) unconscious

Severely underweight:^11^ Weight-for-age *z*-score ≤ 3.0 or a recorded clinician diagnosis of severe malnutrition

Hospital admission: Admission to the district hospital or one of the three rural hospitals in Mchinji district within 2 weeks of study recruitment, as documented by a study data collector at the hospital

SpO_2_: peripheral blood oxygen saturation.^a^ Definitions of moderate hypoxaemia and hypoglycaemia were chosen for this study and are not standard definitions.^b^ We did not extract information on movement as a danger sign and severe chest indrawing was not disaggregated from chest indrawing.^c^ As 5–12 year olds are not included in Integrated Management of Childhood Illness protocols, we used the same danger signs as 2–59-month-olds.

SpO_2_: peripheral blood oxygen saturation.

Notes: Some children referred to hospital from a primary health-care facility did not attend hospital because caregivers did not always follow the referral pathway. Data collectors at primary health-care facilities monitored whether children received onward care at a second primary health-care facility instead.

### Data collection

We employed 20 non-clinical data collectors resident in Mchinji district. They underwent one week’s residential training in study procedures and blood glucose and SpO_2_ measurement. At the end of the training, data collectors were individually assessed on their interpretation of different clinical scenarios to verify their understanding of good-quality SpO_2_ and blood glucose measurements. Clinical staff at all study facilities attended a 2-day refresher training course provided by the district health management team that covered Integrated Management of Childhood Illness protocols. Attendees’ knowledge before and after training was not formally assessed.

Study participants were recruited during standard operating hours (i.e. 08:00 to 15:00, Monday to Friday) from the primary health-care facilities; emergency cases seen outside these times may have been missed. Children were assessed routinely by facility staff who alerted data collectors when a child was referred to another facility (Fig. 1). After obtaining informed consent from caregivers, data collectors measured SpO_2_ with a Lifebox pulse oximeter (Lifebox Foundation, London, England) using the big toe (or finger in children older than 2 years). Then the blood glucose concentration was measured in a capillary sample using Accu-Chek Aviva (Roche, Basel, Switzerland). If severe hypoglycaemia or hypoxaemia was detected, the health-care provider was alerted. Other clinical data were extracted from the child’s health passport or the caregiver’s report and contact details, sociodemographic information and details of care-seeking and treatment were obtained using a brief questionnaire. Enrolment was kept brief and study staff were instructed not to interfere with caregiver decision-making. A unique study barcode sticker was placed inside each recruited child’s health passport.

Outpatients and inpatients at the four referral hospitals and the 14 primary health-care facilities were screened daily by data collectors to document the onward care of recruited children. Both SpO_2_ and blood glucose were measured again by study staff at these locations when a recruited child was identified (Fig. 1). Children were followed up by phone or household visit 2 weeks after hospital discharge or their last confirmed outpatient visit to confirm survival and obtain details of any additional formal or informal care-seeking. A new illness episode was registered if a child presented after the 2-week follow-up period had been completed and, therefore, it was possible for an individual child to be recruited more than once. Verbal autopsies were conducted for children who died using WHO’s 2016 verbal autopsy instrument.^20^ However, because of COVID-19, verbal autopsies were completed for only eight of the 29 children who died.

Data were entered and uploaded daily onto tablet computers using CommCare software (Dimagi Inc., Cambridge, United States of America) and the full list of currently recruited children was visible to all data collectors. Data collectors were supervised by the project manager (a clinical officer), the data manager, and study monitoring and evaluation staff. Problems with implementation were dealt with during frequent supervision visits and monthly group meetings. Ethical approval was obtained from the Research and Ethics Committee at the University of Malawi’s College of Medicine (P.11/18/2538). Caregivers provided informed verbal consent at recruitment and each subsequent interaction.

### Statistical analysis

We recorded hypoxaemia and hypoglycaemia severity at recruitment and compared changes between recruitment and subsequent clinical visits using paired *t*-tests for means and Wilcoxon signed-rank tests for medians. The primary study outcome was the case fatality ratio and the primary exposures of interest were the SpO_2_ level and the blood glucose concentration at recruitment (Box 1). The case fatality ratio was calculated as the number of deaths occurring between recruitment (day 0) and 14 days after hospital discharge or the last confirmed clinical visit divided by the number of children who completed follow-up. Associations with the case fatality ratio were estimated using multivariable Cox proportional hazards models, adjusted for recruitment facility clusters. The survival time was censored at death or 2 weeks after hospital discharge or the last confirmed clinical visit. For children who died on the day of recruitment, the survival time was taken to be 0.5 days. Missing SpO_2_ values at recruitment were included as a distinct category because of previous evidence of an association with mortality.^12^ We adopted the same approach for missing glucose values. Models were adjusted for the presence of general danger signs (Box 1), severe underweight, age, sex and hospital admission.

We were unable to adjust for oxygen or dextrose treatment using multivariable adjustment, interaction terms, stratification or propensity score matching because of confounding by indication (i.e. the most severely ill children were more likely to receive oxygen but were less likely to survive).^21^^,^^22^ However, we conducted exploratory analyses using hospital admission as a proxy for treatment: we performed a stratified analysis by hospital admission and estimated the treatment effect of admission using propensity score matching.^23^ All analyses were performed using Stata v. 14 (StataCorp LLC, College Station, USA).

## Results

**Fig. 2:**
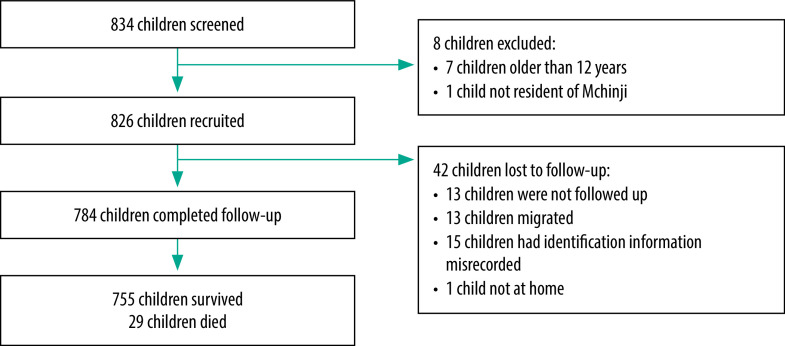
Participant selection and follow-up, Malawi, 2019–2020

Notes: Survival was determined 14 days after hospital discharge or the last clinical visit. An additional three children died after the 14-day follow-up period.

In total, 834 episodes of child illness were screened and 826 children were recruited, of whom 784 (94.9%) completed follow-up (Fig. 2 and Table 1; available from: https://www.who.int/publications/journals/bulletin/). Most follow-ups involved household visits (71.9%; 564/784) and caregivers were contacted on average 1.7 times (range: 1–12). The median follow-up time was 14 days (range: 0–77). The children’s median age was 36 months (interquartile range, IQR: 16–73) and more boys were recruited than girls: 52.9% (437/826) versus 47.1% (389/826), respectively.

**Table 1:** Children’s characteristics at recruitment, prospective cohort study of survival in children with hypoxaemia and/or hypoglycaemia on referral, Malawi, 2019–2020

Variable	No. (%) of children^a^		
	Recruited (*n* = 826)	Completed follow-up (*n* = 784)	Lost to follow-up (*n* = 42)
**Demographic**			
Age			
< 2 months	38 (4.6)	38 (4.9)	0 (0.0)
2–11 months	105 (12.7)	98 (12.5)	7 (16.7)
12–59 months	413 (50.0)	390 (49.7)	23 (54.8)
5–12 years	270 (32.7)	258 (32.9)	12 (28.6)
Sex			
Male	437 (52.9)	419 (53.4)	18 (42.9)
Female	389 (47.1)	365 (46.6)	24 (57.1)
**Socioeconomic**			
Mother’s age in years, mean (SD)	28.9 (7.8)	28.9 (7.7)	28.9 (8.9)
Maternal education			
None	79 (9.6)	73 (9.3)	6 (14.3)
Primary	659 (79.8)	624 (79.6)	35 (83.3)
Secondary or further	85 (10.3)	85 (10.8)	0 (0.0)
Missing data	3 (0.4)	2 (0.3)	1 (2.4)
Maternal marital status			
Married	703 (85.1)	664 (84.7)	39 (92.9)
Not married	122 (14.8)	119 (15.2)	3 (7.1)
Missing data	1 (0.1)	1 (0.1)	0 (0.0)
**Clinical**			
SpO_2_			
Mean value (SD), %	94.9 (5.9)	94.9 (5.9)	94.4 (6.5)
Normoxaemia^b^	637 (77.1)	605 (77.2)	32 (76.2)
Moderate hypoxaemia^b^	108 (13.1)	104 (13.3)	4 (9.5)
Severe hypoxaemia^b^	71 (8.6)	65 (8.3)	6 (14.3)
Missing data^c^	10 (1.2)	10 (1.3)	0 (0.0)
Blood glucose			
Mean concentration (SD), mmol/L	5.8 (2.0)	5.8 (2.0)	5.8 (2.4)
Normoglycaemia^b^	725 (87.8)	687 (87.6)	38 (90.5)
Moderate hypoglycaemia^b^	74 (9.0)	71 (9.1)	3 (7.1)
Severe hypoglycaemia^b^	19 (2.3)	18 (2.3)	1 (2.4)
Missing data^d^	8 (1.0)	8 (1.0)	0 (0.0)
Routine diagnosis^e,f^			
Acute respiratory infection or pneumonia	111 (13.6)	105 (13.5)	6 (14.6)
Malaria	313 (38.3)	301 (38.7)	12 (29.3)
Sepsis or meningitis	92 (11.3)	86 (11.1)	6 (14.6)
Diarrhoea	18 (2.2)	18 (2.3)	0 (0.0)
Fever (unclassified)	39 (4.8)	38 (4.9)	1 (2.4)
Skin condition	39 (4.8)	37 (4.8)	2 (4.9)
Malnutrition	36 (4.4)	34 (4.4)	2 (4.9)
Anaemia	55 (6.7)	53 (6.8)	2 (4.9)
Trauma	201 (24.6)	193 (24.8)	8 (19.5)
Other infectious condition	13 (1.6)	12 (1.5)	1 (2.4)
Other non-infectious condition	109 (13.3)	101 (13.0)	8 (19.5)

SD: standard deviation; SpO_2_: peripheral blood oxygen saturation.

^a^ All values in the table represent absolute numbers and percentages unless otherwise stated.

^b^ Definitions of normoxaemia, hypoxaemia, normoglycaemia and hypoglycaemia are given in Box 1.

^c^ Reasons for missing data were: (i) six children too agitated (6); (ii) three children unconscious and receiving care; and (iii) a biologically plausible value could not be obtained for one child.

^d^ Reasons for missing data were: (i) no test strips available (one child); (ii) no lancet available (two children); (iii) glucometer not working (four children); and (iv) transport for referral was found before the test could be completed (one child).

^e^ Diagnosis made by the health-care provider at recruitment.

^f^ Children could receive more than one diagnosis.

We recorded 29 deaths within 2 weeks of hospital discharge or the last confirmed clinical visit, which gave a case fatality ratio of 3.7% (29/784). We recruited 13 children more than once, of whom two died (15.4%). The case fatality ratio was highest in infants younger than 2 months (15.8%; 6/38) and lowest in children aged 5 to 12 years (2.3%; 6/258; *P*-value: < 0.001). No significant difference in case fatality ratio was observed by sex (*P*-value: 0.850). The median time from recruitment to death was 1 day (IQR: 0–4) and 44.8% (13/29) of deaths occurred within 24 hours. Seven of the 13 children who died on the day of recruitment were not admitted (available in data repository).^24^ Following recruitment, 37.1% (306/826) of children were admitted to hospital and 41.7% (344/826) attended another facility.

### Hypoxaemia

Overall, 8.6% (71/826) of children were severely hypoxaemic at recruitment and 13.1% (108/826) were moderately hypoxaemic (Table 1). Severe hypoxaemia was significantly more frequent in children younger than 2 months (28.9%; 11/38) than in those aged 5 to 12 years (4.1%; 11/270; *P*-value: < 0.001). The case fatality ratio among children who completed follow-up was 13.9% (9/65) in those with severe hypoxaemia, 3.9% (4/104) in those with moderate hypoxaemia and 2.3% (14/605) in those with normoxaemia. Among all severely hypoxaemic children (Table 2), the most frequent diagnoses were acute respiratory infection (45.1%; 32/71) and malaria (39.4%; 28/71). Only 24.0% (17/71) of hypoxaemic children had a documented respiratory rate but chest indrawing was common: 42.3% (30/71) of severely hypoxaemic children and 33.3% (36/108) of moderately hypoxaemic children had this clinical sign.

**Table 2:** Children’s characteristics at recruitment, by blood oxygen level, prospective cohort study of survival in children with hypoxaemia and/or hypoglycaemia on referral, Malawi, 2019–2020

Variable	No. (%) of children			
	Normoxaemic^a^ (*n* = 637)	Moderately hypoxaemic^a^ (*n* = 108)	Severely hypoxaemic^a^ (*n* = 71)	Missing data (*n* = 10)
**Demographic characteristic**				
Age				
< 2 months	18 (2.8)	7 (6.5)	11 (15.5)	2 (20.0)
2–11 months	60 (9.4)	22 (20.4)	20 (28.2)	3 (30.0)
12–59 months	310 (48.7)	69 (63.9)	29 (40.9)	5 (50.0)
5–12 years	249 (39.1)	10 (9.3)	11 (15.5)	0 (0.0)
Sex				
Male	339 (53.2)	57 (52.8)	37 (52.1)	4 (40.0)
Female	298 (46.8)	51 (47.2)	34 (47.9)	6 (60.0)
**Clinical characteristic**				
Fast breathing^b^				
Not present	76 (11.9)	18 (16.7)	6 (8.5)	2 (20.0)
Present	51 (8.0)	8 (7.4)	11 (15.5)	0 (0.0)
Missing data	510 (80.1)	82 (75.9)	54 (76.1)	8 (80.0)
Temperature, °C				
< 35.5	27 (4.2)	1 (0.9)	2 (2.8)	1 (10.0)
35.5–37.4	317 (49.8)	45 (41.7)	27 (38.0)	5 (50.0)
≥ 37.5	202 (31.7)	55 (50.9)	35 (49.3)	4 (40.0)
Missing data	91 (14.3)	7 (6.5)	7 (9.9)	0 (0.0)
Malaria status				
mRDT-positive	205 (32.2)	37 (34.3)	28 (39.4)	2 (20.0)
mRDT-negative	60 (9.4)	17 (15.7)	14 (19.7)	2 (20.0)
No mRDT result	372 (58.4)	54 (50.0)	29 (40.9)	6 (60.0)
Chest indrawing^c^				
Not present	576 (90.4)	72 (66.7)	40 (56.3)	8 (80.0)
Present	60 (9.4)	36 (33.3)	30 (42.3)	2 (20.0)
Missing data	1 (0.2)	0 (0.0)	1 (1.4)	0 (0)
Danger signs^d^				
Not present	325 (51.0)	22 (20.4)	11 (15.5)	4 (40.0)
Present	312 (49.0)	86 (79.6)	60 (84.5)	6 (60.0)
Severely underweight				
No	546 (85.7)	88 (81.5)	60 (84.5)	8 (80.0)
Yes	91 (14.3)	20 (18.5)	11 (15.5)	2 (20.0)
Routine diagnosis^e,f^				
Acute respiratory infection or pneumonia	45 (7.1)	31 (28.7)	32 (45.1)	3 (30.0)
Malaria	229 (36.0)	53 (49.1)	28 (39.4)	3 (30.0)
Sepsis or meningitis	63 (9.9)	17 (15.7)	12 (16.9)	0 (0.0)
Diarrhoea	14 (2.2)	3 (2.8)	1 (1.4)	0 (0.0)
Fever (unclassified)	22 (3.5)	11 (10.2)	6 (8.5)	0 (0.0)
Skin condition	33 (5.2)	5 (4.6)	0 (0.0)	1 (10.0)
Malnutrition	28 (4.4)	6 (5.6)	2 (2.8)	0 (0.0)
Anaemia	40 (6.3)	8 (7.4)	7 (9.9)	0 (0.0)
Trauma	190 (29.8)	6 (5.6)	4 (5.6)	1 (10.0)
Other infectious condition	12 (1.9)	1 (0.9)	0 (0.0)	0 (0.0)
Other non-infectious condition	89 (14.0)	6 (5.6)	12 (16.9)	2 (20.0)

mRDT: malaria rapid diagnostic test.

^a^ Definitions of normoxaemia and hypoxaemia are given in Box 1.

^b^ Fast breathing was a rate ≥ 60 breaths/min in children aged < 2 months, ≥ 50 breaths/min in those aged 2–11 months, ≥ 40 breaths/min in those aged 12–59 months (World Health Organization Integrated Management of Childhood Illness 2014 guidelines)^1^ and ≥ 30 breaths/min in those aged 5–12 years (World Health Organization Integrated Management of Adolescent and Adult Illness 2012 guidelines).^25^

^c^ Severe chest indrawing in children aged < 2 months.

^d^ Danger signs are described in Box 1.

^e^ Diagnosis made by the health-care provider at recruitment.

^f^ Children could receive more than one diagnosis.

After recruitment, 63.4% (45/71) of severely hypoxaemic children, 50.9% (55/108) of moderately hypoxaemic children and 37.5% (239/637) of normoxaemic children attended another facility (Table 3; available from: https://www.who.int/publications/journals/bulletin/). The median SpO_2_ increased after recruitment in both those with severe hypoxaemia (from 84% to 92%; *P*-value: < 0.001) and moderate hypoxaemia (from 92% to 95%; *P*-value: 0.006).

**Table 3:** Care-seeking and clinical progression after recruitment, by blood oxygen level, prospective cohort study of survival in children with hypoxaemia and/or hypoglycaemia on referral, Malawi, 2019–2020

Group	No. in group	Children who received further care,^a^ no. (%)	Hours to receipt of further care,^b^ median (IQR)	SpO_2_, %		
				Median (IQR)		*P* ^c^
				At study recruitment	At subsequent facility	
All children	826	344 (41.7)	5.0 (2.9–8.0)	97 (94–98)	97 (95–98)	0.060
Normoxaemic children^d^	637	239 (37.5)	5.2 (3.3–11.3)	98 (96–98)	97 (95–98)	0.121
Moderately hypoxaemic children^d^	108	55 (50.9)	4.2 (3.2–7.0)	92 (91–93)	95 (90–97)	0.006
Severely hypoxaemic children^d^	71	45 (63.4)	3.3 (2.0–5.4)	84 (75–87)	92 (87–96)	< 0.001
Children with missing data	10	5 (50.0)	17.6 (7.1–28.3)	ND	96 (95–96)	NA

IQR: interquartile range; NA: not applicable; ND: not determined; SpO_2_: peripheral blood oxygen saturation.

^a^ Further care included both hospital admission (306 children) and outpatient care at a hospital or health-care facility (38 children).

^b^ The time from recruitment to presentation at the first subsequent facility.

^c^ Medians were compared using the Wilcoxon signed-rank test.

^d^ Definitions of normoxaemia and hypoxaemia are given in Box 1.

**Fig. 3:**
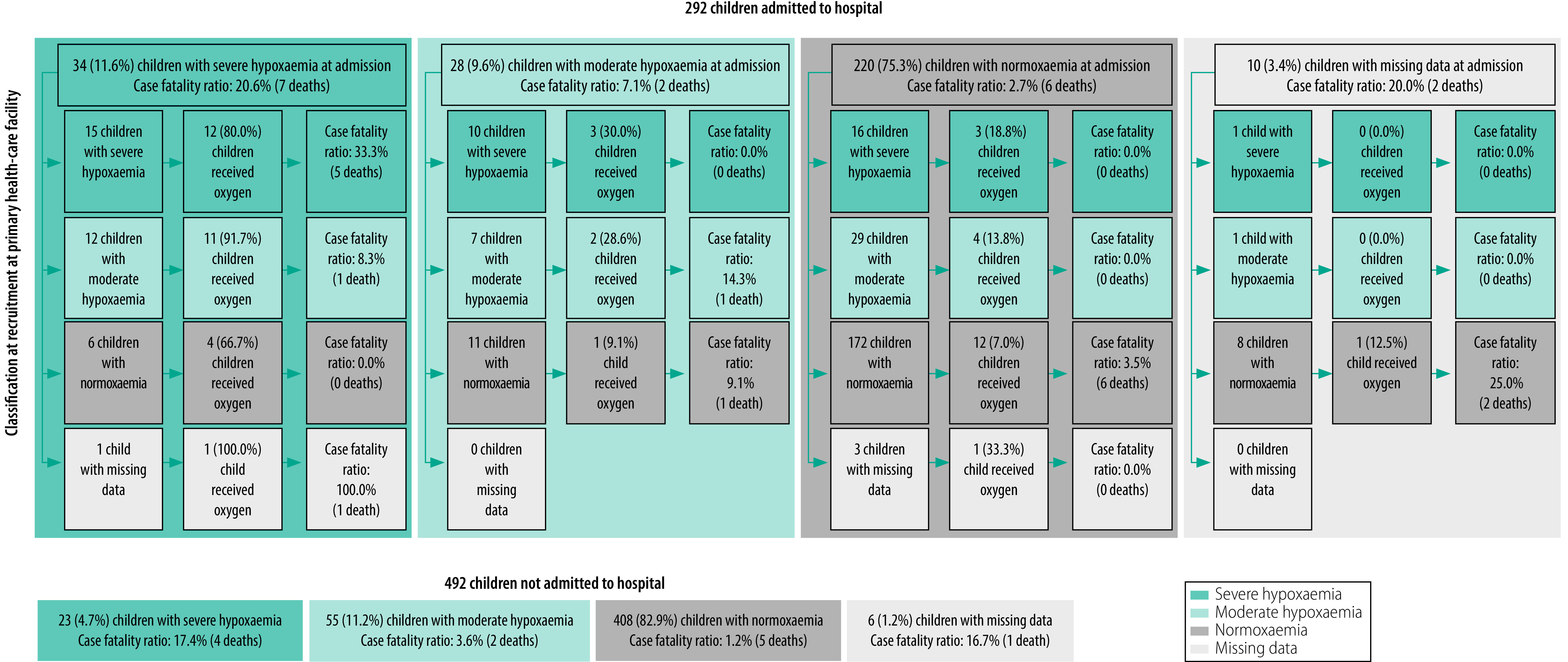
Case fatality ratio and oxygen treatment, by blood oxygen level at recruitment and hospital admission, prospective cohort study of survival in children with hypoxaemia and/or hypoglycaemia on referral, Malawi, 2019–2020

Note: Definitions of hypoxaemia and normoxaemia are given in Box 1.

Of the 292 children who were admitted to hospital and completed follow-up, 34 (11.6%) were severely hypoxaemic on arrival (Fig. 3); 28 of the 34 (82.4%) received oxygen. Of the 49 children with moderate hypoxaemia who were admitted, 12 (24.5%) had progressed to severe hypoxaemia and one of the 12 died (case fatality ratio: 8.3%) – this child did not receive oxygen. Of the 42 children with severe hypoxaemia at recruitment who were admitted, 15 (35.7%) remained severely hypoxaemic on arrival at hospital and five of the 15 died (case fatality ratio: 33.3%). The case fatality ratio for children with an SpO_2_ below 90% at hospital admission was 20.6% (7/34), which was similar to the ratio for severely hypoxaemic children who were not admitted (17.4%; 4/23).

### Hypoglycaemia

Overall, 2.3% (19/826) of children were severely hypoglycaemic at recruitment and 9.0% (74/826) were moderately hypoglycaemic (Table 1). The case fatality ratio among children who completed follow-up was 27.8% (5/18) in those with severe hypoglycaemia, 5.6% (4/71) in those with moderate hypoglycaemia and 2.9% (20/687) in those with normoglycaemia. Of the 19 severely hypoglycaemic children overall (Table 4), 15 (79.0%) presented with a danger sign and the most frequent diagnoses were malaria (52.6%; 10/19), malnutrition (31.6%; 6/19) and sepsis or meningitis (21.1%; 4/19). Severe hypoglycaemia was more frequent in girls than boys: 3.1% (12/389) versus 1.6% (7/437), respectively (*P*-value: 0.160). 

**Table 4:** Children’s characteristics at recruitment, by blood glucose concentration, prospective cohort study of survival in children with hypoxaemia and/or hypoglycaemia on referral, Malawi, 2019–2020

Variable	No. (%) of children			
	Normoglycaemic^a^ (*n* = 725)	Moderately hypoglycaemic^a^ (*n* = 74)	Severely hypoglycaemic^a^ (*n* = 19)	Missing data (*n* = 8)
**Demographic characteristic**				
Age				
< 2 months	32 (4.4)	4 (5.4)	1 (5.3)	1 (12.5)
2–11 months	96 (13.2)	4 (5.4)	3 (15.8)	2 (25.0)
12–59 months	358 (49.4)	38 (51.4)	13 (68.4)	4 (50.0)
5–12 years	239 (33.0)	28 (37.8)	2 (10.5)	1 (12.5)
Sex				
Male	387 (53.4)	36 (48.7)	7 (36.8)	7 (87.5)
Female	338 (46.6)	38 (51.4)	12 (63.2)	1 (12.5)
**Clinical characteristic**				
Fast breathing^b^				
Not present	93 (12.8)	5 (6.8)	4 (21.1)	0 (0.0)
Present	57 (7.9)	11 (14.9)	2 (10.5)	0 (0.0)
Missing data	575 (79.3)	58 (78.4)	13 (68.4)	8 (100.0)
Temperature, °C				
< 35.5	26 (3.6)	2 (2.7)	2 (10.5)	1 (12.5)
35.5–37.4	340 (46.9)	40 (54.1)	11 (57.9)	3 (37.5)
≥ 37.5	266 (36.7)	23 (31.1)	5 (26.3)	2 (25.0)
Missing data	93 (12.8)	9 (12.2)	1 (5.3)	2 (25.0)
Malaria status				
mRDT-positive	244 (33.7)	21 (28.4)	5 (26.3)	2 (25.0)
mRDT-negative	79 (10.9)	9 (12.2)	4 (21.1)	1 (12.5)
No mRDT result	402 (55.5)	44 (59.5)	10 (52.6)	5 (62.5)
Chest indrawing^c^				
Not present	613 (84.6)	61 (82.4)	15 (79.0)	7 (87.5)
Present	112 (15.5)	12 (16.2)	3 (15.8)	1 (12.5)
Missing data	0 (0.0)	1 (1.4)	1 (5.3)	0 (0.0)
Danger signs^d^				
Not present	323 (44.5)	32 (43.2)	4 (21.0)	3 (37.5)
Present	402 (55.5)	42 (56.8)	15 (79.0)	5 (62.5)
Severely underweight				
No	636 (87.7)	51 (68.9)	9 (47.4)	6 (75.0)
Yes	89 (12.3)	23 (31.1)	10 (52.6)	2 (25.0)
Routine diagnosis^e,f^				
Acute respiratory infection or pneumonia	100 (13.8)	6 (8.1)	3 (15.8)	2 (25.0)
Malaria	274 (37.8)	27 (36.5)	10 (52.6)	2 (25.0)
Sepsis or meningitis	74 (10.2)	13 (17.6)	4 (21.1)	1 (12.5)
Diarrhoea	15 (2.1)	1 (1.4)	1 (5.3)	1 (12.5)
Fever (unclassified)	32 (4.4)	6 (8.1)	0 (0.0)	1 (12.5)
Skin condition	38 (5.2)	1 (1.4)	0 (0.0)	0 (0.0)
Malnutrition	21 (2.9)	9 (12.2)	6 (31.6)	0 (0.0)
Anaemia	44 (6.1)	8 (10.8)	2 (10.5)	1 (12.5)
Trauma	189 (26.1)	11 (14.9)	0 (0.0)	1 (12.5)
Other infectious condition	11 (1.5)	2 (2.7)	0 (0.0)	0 (0.0)
Other non-infectious condition	91 (12.6)	13 (17.6)	3 (15.8)	2 (25.0)

mRDT: malaria rapid diagnostic test.

^a^ Definitions of normoglycaemia and hypoglycaemia are given in Box 1.

^b^ Fast breathing was a rate ≥ 60 breaths/min in children aged < 2 months, ≥ 50 breaths/min in those aged 2–11 months, ≥ 40 breaths/min in those aged 12–59 months (World Health Organization Integrated Management of Childhood Illness 2014 guidelines)^1^ and ≥ 30 breaths/min in those aged 5–12 years (World Health Organization Integrated Management of Adolescent and Adult Illness 2012 guidelines).^25^

^c^ Severe chest indrawing in children aged < 2 months.

^d^ Danger signs are described in Box 1.

^e^ Diagnosis made by the health-care provider at recruitment.

^f^ Children could receive more than one diagnosis.

Presentation at another facility after recruitment (Table 5; available from: https://www.who.int/publications/journals/bulletin/) was more frequent for children with severe hypoglycaemia (57.9%; 11/19) than for those with moderate hypoglycaemia (40.5%; 30/74) or normoglycaemia (41.2%; 299/725). Although there was no difference in the mean blood glucose concentration between recruitment and arrival at another facility overall, the mean was significantly higher on subsequent measurement for both severely and moderately hypoglycaemic children (Table 5). No severely or moderately hypoglycaemic child was given pre-referral glucose treatment at recruitment.

**Table 5:** Care-seeking and clinical progression after recruitment, by blood glucose concentration, prospective cohort study of survival in children with hypoxaemia and/or hypoglycaemia on referral, Malawi, 2019–2020

Group	No. in group	Children who received further care,^a^ no. (%)	Hours to receipt of further care,^b^ median (IQR)	Blood glucose concentration, mmol/L		
				Mean (95% CI)		*P* ^c^
				At study recruitment	At subsequent facility	
All children	826	344 (41.7)	5.0 (2.9–8.0)	5.92 (5.70 to 6.14)	5.86 (5.66 to 6.06)	0.603
Normoglycaemic children^d^	725	299 (41.2)	5.0 (3.1–7.9)	6.31 (6.10 to 6.52)	6.03 (5.82 to 6.24)	0.018
Moderately hypoglycaemic children^d^	74	30 (40.5)	4.3 (3.2–25.7)	3.48 (3.33 to 3.61)	4.75 (4.18 to 5.32)	< 0.001
Severely hypoglycaemic children^d^	19	11 (57.9)	3.9 (2.3–7.1)	2.39 (2.15 to 2.63)	4.48 (3.35 to 5.62)	0.001
Children with missing data	8	4 (50.0)	3.8 (2.9–6.6)	ND	7.17 (4.06 to 10.27)	NA

CI: confidence interval; IQR: interquartile range; NA: not applicable; ND: not determined.

^a^ Further care included both hospital admission (306 children) and outpatient care at a hospital or health-care facility (38 children).

^b^ The time from recruitment to presentation at the first subsequent facility.

^c^ Means were compared using a *t*-test.

^d^ Definitions of normoglycaemia and hypoglycaemia are given in Box 1.

**Fig. 4:**
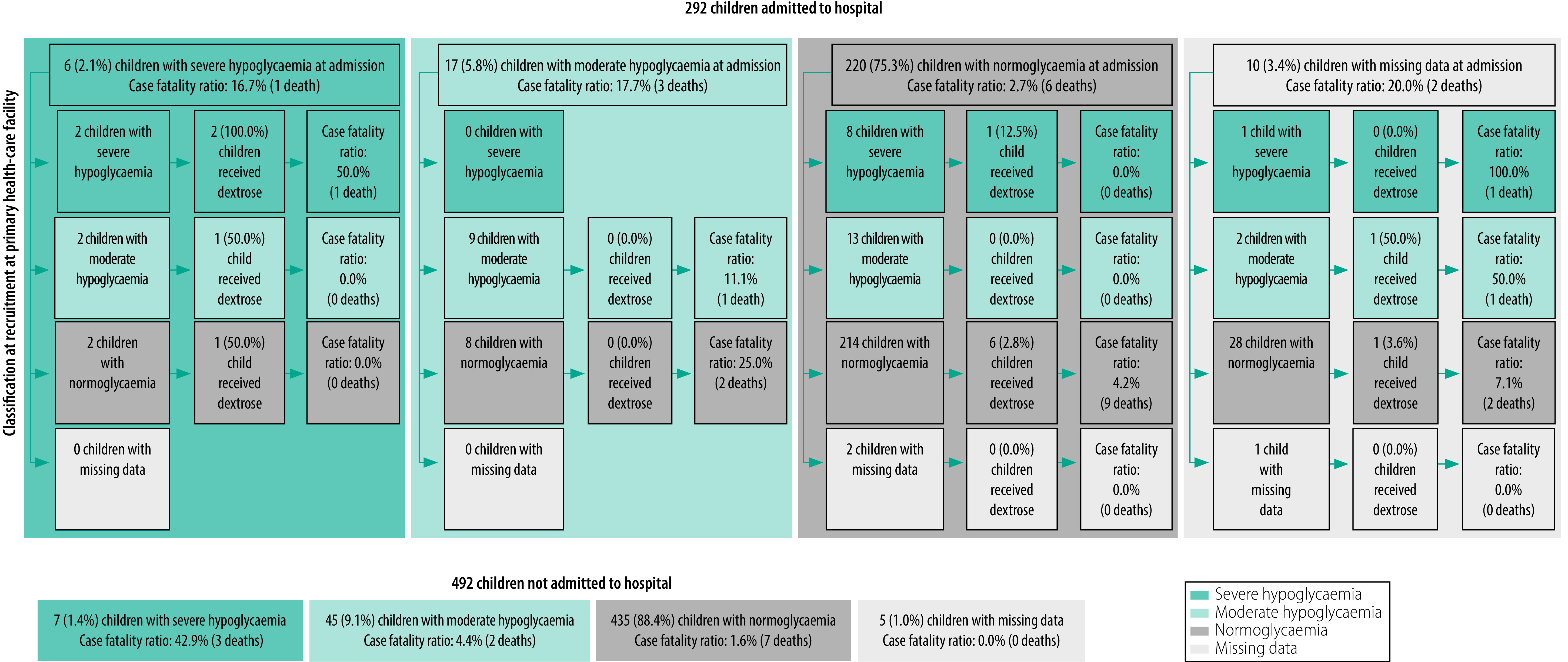
Case fatality ratio and dextrose treatment, by blood glucose concentration at recruitment and hospital admission, prospective cohort study of survival in children with hypoxaemia and/or hypoglycaemia on referral, Malawi, 2019–2020

Note: Definitions of hypoglycaemia and normoglycaemia are given in Box 1.

Of the 292 children admitted to hospital, six (2.1%) had severe hypoglycaemia at admission; four of the six (66.7%) received dextrose treatment (Fig. 4). Of the 26 children with moderate hypoglycaemia at recruitment who were subsequently admitted, two (7.7%) had severe hypoglycaemia at admission and 13 (50.0%) had a normal glucose level. The case fatality ratio was similar among children who had moderate or severe hypoglycaemia at admission: 17.7% (3/17) versus 16.7% (1/6), respectively. However, the ratio was 42.9% (3/7) among severely hypoglycaemic children who were not admitted.

### Survival and treatment effects

The results of the adjusted Cox proportional hazards model for survival are presented in Table 6. Both severe hypoxaemia (adjusted hazard ratio, aHR, compared with normoxaemia: 4.05; 95% confidence interval, CI: 1.65 to 9.94) and severe hypoglycaemia (aHR compared with normoglycaemia: 7.60; 95% CI: 2.07 to 27.92) at recruitment were independently associated with death. There was no significant association with either moderate hypoxaemia or moderate hypoglycaemia.

**Table 6:** Factors associated with death, adjusted Cox proportional hazards model, prospective cohort study of survival in children with hypoxaemia and/or hypoglycaemia on referral, Malawi, 2019–2020

Factor	Hazard of death^a,b^	
	aHR (95% CI)^c^	*P*
**Blood oxygen level**		
Normoxaemia^d^	Reference	NA
Moderate hypoxaemia^d^	1.27 (0.40 to 3.97)	0.648
Severe hypoxaemia^d^	4.05 (1.65 to 9.94)	0.002
Missing data	1.84 (0.24 to 14.08)	0.559
**Blood glucose concentration**		
Normoglycaemia^d^	Reference	NA
Moderate hypoglycaemia^d^	2.04 (0.54 to 7.64)	0.291
Severe hypoglycaemia^d^	7.60 (2.07 to 27.92)	0.002
Missing data^e^	ND	ND
**Danger signs^f^**		
No	Reference	NA
Yes	2.51 (0.84 to 7.50)	0.098
**Severely underweight**		
No	Reference	NA
Yes	1.45 (0.67 to 3.18)	0.347
**Hospital admission**		
No	Reference	NA
Yes	1.20 (0.53 to 2.73)	0.659
**Sex**		
Male	Reference	NA
Female	1.19 (0.50 to 2.84)	0.700
**Age**		
5–12 years	Reference	NA
12–59 months	0.72 (0.22 to 2.32)	0.579
2–11 months	1.07 (0.28 to 4.05)	0.924
< 2 months	2.98 (0.68 to 13.12)	0.149

aHR: adjusted hazard ratio; CI: confidence interval; NA: not applicable; ND: not determined.

^a^ The analysis included data on 776 children.

^b^ The hazard of death between study recruitment and 14 days after hospital discharge or the last clinical visit.

^c^ The proportional hazards assumption was tested using Schoenfeld residuals and was found not to be violated (*P*-value: 0.201).

^d^ Definitions of normoxaemia, hypoxaemia, normoglycaemia and hypoglycaemia are given in Box 1.

^e^ As all eight children with missing data survived, they were dropped from the model because of perfect prediction.

^f^ Danger signs are described in Box 1.

In the analysis in which children were stratified by hospital admission, admission appeared to decrease the hazard of death for both those with severe hypoxaemia and those with severe hypoglycaemia. Among children with severe hypoxaemia, the aHR for death compared with normoxaemia was 9.14 in those who were not admitted versus 2.34 in those who were. Among children with severe hypoglycaemia, the aHR for death compared with normoglycaemia was 15.74 in those who were not admitted versus 4.12 in those who were. However, the CIs for these hazard ratios were wide (available in the data repository).^24^ Overall, the treatment effect of hospital admission was estimated to be a 1.39% (95% CI: −6.81 to 4.02) reduction in the case fatality ratio among those admitted (data repository).^24^ The estimated effect was larger for children with moderate or severe hypoxaemia but was not significant.

## Discussion

We found that severe hypoxaemia and severe hypoglycaemia were significantly associated with death among children referred from primary health-care facilities to hospitals in Malawi. Although neither moderate hypoxaemia nor moderate hypoglycaemia was significantly associated with increased mortality, our exploratory analyses suggested that hospital admission may decrease the risk. Only 37% (292/784) of children in the study were admitted to hospital and, overall, more than 95% of those with moderate hypoxaemia or hypoglycaemia who were not admitted survived, irrespective of the presence of danger signs. However, over a quarter of referred children with severe hypoglycaemia died and these deaths predominantly occurred within 24 hours, which suggests that the severity of the disease may have been recognized late and care-seeking may have been delayed, as has been observed in previous studies in Malawi.^5^^,^^26^

The Integrated Management of Childhood Illness protocol recommends presumptive hypoglycaemia treatment before referral.^1^ In 2019, the majority of facilities in Mchinji had a glucometer and a stock of dextrose.^27^ Although it would be unreasonable to expect that blood glucose measurements would be carried out routinely at our study facilities as they are not included in the protocol, the fact that no hypoglycaemic child reportedly received presumptive glucose treatment points to a gap in the protocol’s implementation. Nevertheless, we observed an increase in the mean glucose concentration after recruitment in both moderately and severely hypoglycaemic children, which suggests that health-care providers may have given caregivers advice on feeding. Alternatively, many of the most acutely hypoglycaemic children may have died before hospital admission. Our findings support the use of presumptive glucose treatment. However, greater efforts must be made to ensure this happens, along with subsequent glucose monitoring and management.^28^

In the Integrated Management of Childhood Illness algorithm for respiratory infections, an SpO_2_ below 90% is an indication for referral.^1^ In agreement with previous reports,^29^ we found that hypoxaemia was relatively common, even in the absence of pneumonia. Although functional oximeters were reportedly available in 29.8% (14/47) of sampled facilities in Malawi,^30^ health-care workers often made referral decisions without using pulse oximetry. We found that 28.9% of infants younger than 2 months were severely hypoxaemic, similar to the 22.6% (53/235) reported in a previous study from Malawi.^31^ The quality of oximetry measurements in these infants can be poor due to badly fitting probes, non-cooperation or perfusion issues. However, reported diagnoses were consistent with conditions where hypoxaemia was expected (e.g. congenital heart disease, asphyxia, apnoea, pneumonia and sepsis). Given the role of pulse oximetry in detecting congenital heart disease in neonates,^32^ which is often asymptomatic, neonatal SpO_2_ measurements must be feasible and reliable. More broadly, we observed that respiratory rates were rarely documented, which corresponds with previous findings that respiratory examinations are often poorly conducted in Malawi.^33^^–^^36^ There is, therefore, a need to improve pneumonia diagnosis and management.

We found that both moderate hypoxaemia and moderate hypoglycaemia at recruitment were associated with a non-significant increase in the hazard of death among children, which contradicts previous hospital-based studies.^7^^,^^8^^,^^14^^,^^31^ Moreover, our exploratory analysis, though it lacked statistical power, suggested that hospitalization may have reduced mortality in these patient groups. Strikingly, 11 of the 12 children with moderate hypoxaemia who progressed to severe hypoxaemia by hospital admission survived – the child who died did not receive oxygen. In contrast, a third of children admitted with persistently severe hypoxaemia died, even though most received oxygen. These findings suggest that earlier identification and prompt care-seeking could reduce mortality.^37^ However, given confounding by indication (i.e. the most severely ill children are more likely to receive oxygen but also to die) and potential survivorship bias (i.e. children have to survive long enough to reach hospital), well-designed trials are needed to provide evidence for guideline reviews.

The influence of dextrose treatment on survival was less clear. Although moderate hypoglycaemia at recruitment was not a significant risk factor for death, the case fatality ratio in children with moderate hypoglycaemia at hospital admission was 17.7%, higher than for any other admission hypoglycaemia category. The recent SugarFACT trial in Malawi failed to show that treatment improved survival in children with hypoglycaemia,^18^ which reinforces the need for better understanding of the management of these patients. Our observation that blood glucose and SpO_2_ categories changed between recruitment and hospitalization in most children raises the important question of whether serial measurements are preferable to one-off spot checks for case management and for identifying the need for urgent care and outpatient monitoring.^38^

Although few infants younger than 2 months were recruited, they had the highest case fatality ratio of all age groups. We were surprised to find that 32.7% (270/826) of children recruited were aged 5 to 12 years and that their case fatality ratio was comparable to that of children aged 12 to 59 months: 2.3% (6/258) versus 3.1% (12/390), respectively. This older age group is overlooked, being neither explicitly included in an Integrated Management of Childhood Illness chart booklet nor targeted by sustainable development goals.^39^ Moreover, measurement of SpO_2_ and blood glucose levels do not appear to be informative for this age group and more research is warranted.

Our study had three key limitations. First, because of the COVID-19 pandemic, we stopped recruitment before the planned closure date and verbal autopsies were not completed for all deaths. During follow-ups and verbal autopsies, we asked about care-seeking to validate data collection at facilities. Given that the response rate varied by survival status, we chose not to use these data and it is possible, therefore, that we were not able to confirm all instances of onward care. To minimize the possibility that children admitted out of hours were missed, hospital-based data collectors reviewed patient charts each morning. Nevertheless, children who presented to primary-care facilities out of hours would have been missed, resulting in lower recruitment and the under-ascertainment of onward care. Second, we used non-clinical data collectors and it is plausible that some of the variation in hypoxaemia category between recruitment and subsequent care resulted from measurement quality issues as oximetry in young infants requires skill. Finally, we relied on routine clinical assessment and decision-making by health-care workers for deciding on study eligibility and it is possible that some hypoxaemic children who should have been referred were missed. We were unable to validate key clinical variables and problems with routine data quality were apparent (e.g. the absence of respiratory rate data) despite Integrated Management of Childhood Illness refresher training.

Mortality among children with severe hypoxaemia or hypoglycaemia who were referred from primary care in Malawi was high. For hypoglycaemia, our findings support current recommendations for presumptive glucose treatment but further research is needed to determine the optimal threshold for treatment and the best management for this group. For hypoxaemia, timely care-seeking, routine pulse oximetry, and earlier identification and referral of severely hypoxaemic children could reduce the risk of death. However, given that most referred children in our study were not subsequently admitted to hospital but survived, greater understanding of how best to manage moderately hypoxaemic children is needed. Optimal management must take into account the burden placed by referral on the health system and on patients as well as the clinical benefits of treatment.

## References

[1] Integrated Management of Childhood Illness [internet]. Geneva: World Health Organization; 2022. Available from: https://www.who.int/teams/maternal-newborn-child-adolescent-health-and-ageing/child-health/integrated-management-of-childhood-illness [cited 2022 Feb 4].

[2] WHO/UNICEF joint statement. Integrated community case management (iCCM). Geneva & New York: World Health Organization & United Nations Children’s Fund; 2012. Available from: https://www.who.int/maternal_child_adolescent/documents/statement_child_services_access_whounicef.pdf [cited 2022 Feb 4].

[3] Integrated Management of Childhood Illness: management of the sick young infant aged up to 2 months: IMCI chart booklet. Geneva: World Health Organization; 2019. Available from: https://apps.who.int/iris/handle/10665/326448 [cited 2022 Feb 4].

[4] Nolan T, Angos P, Cunha AJ, Muhe L, Qazi S, Simoes EA, et al. Quality of hospital care for seriously ill children in less-developed countries. Lancet. 2001 Jan 13;357(9250):106–10. 10.1016/S0140-6736(00)03542-X 11197397

[5] King C, Banda M, Bar-Zeev N, Beard J, French N, Makwenda C, et al. Care-seeking patterns amongst suspected paediatric pneumonia deaths in rural Malawi. Gates Open Res. 2021 May 6;4(178):178. 10.12688/gatesopenres.13208.2 33537557 PMC7835598

[6] Peterson S, Nsungwa-Sabiiti J, Were W, Nsabagasani X, Magumba G, Nambooze J, et al. Coping with paediatric referral – Ugandan parents’ experience. Lancet. 2004 Jun 12;363(9425):1955–6. 10.1016/S0140-6736(04)16411-8 15194257

[7] Ngwalangwa F, Phiri CHA, Dube Q, Langton J, Hildenwall H, Baker T. Risk factors for mortality in severely ill children admitted to a tertiary referral hospital in Malawi. Am J Trop Med Hyg. 2019 Sep;101(3):670–5. 10.4269/ajtmh.19-0127 31287044 PMC6726928

[8] Nadjm B, Mtove G, Amos B, Hildenwall H, Najjuka A, Mtei F, et al. Blood glucose as a predictor of mortality in children admitted to the hospital with febrile illness in Tanzania. Am J Trop Med Hyg. 2013 Aug;89(2):232–7. 10.4269/ajtmh.13-0016 23817332 PMC3741242

[9] Achoki R, Opiyo N, English M. Mini-review: management of hypoglycaemia in children aged 0–59 months. J Trop Pediatr. 2010 Aug;56(4):227–34. 10.1093/tropej/fmp109 19933785 PMC2948531

[10] Lazzerini M, Sonego M, Pellegrin MC. Hypoxaemia as a mortality risk factor in acute lower respiratory infections in children in low and middle-income countries: systematic review and meta-analysis. PLoS One. 2015 Sep 15;10(9):e0136166. 10.1371/journal.pone.0136166 26372640 PMC4570717

[11] WHO child growth standards: length/height-for-age, weight-for-age, weight-for-length, weight-for-height and body mass index-for-age: methods and development. Geneva: World Health Organization; 2006. Available from: https://www.who.int/publications/i/item/924154693X [cited 2022 Feb 4].

[12] Colbourn T, King C, Beard J, Phiri T, Mdala M, Zadutsa B, et al. Predictive value of pulse oximetry for mortality in infants and children presenting to primary care with clinical pneumonia in rural Malawi: a data linkage study. PLoS Med. 2020 Oct 23;17(10):e1003300. 10.1371/journal.pmed.1003300 33095763 PMC7584207

[13] McCollum ED, King C, Deula R, Zadutsa B, Mankhambo L, Nambiar B, et al. Pulse oximetry for children with pneumonia treated as outpatients in rural Malawi. Bull World Health Organ. 2016 Dec 1;94(12):893–902. 10.2471/BLT.16.173401 27994282 PMC5153930

[14] Hooli S, Colbourn T, Lufesi N, Costello A, Nambiar B, Thammasitboon S, et al. Predicting hospitalised paediatric pneumonia mortality risk: an external validation of RISC and mRISC, and local tool development (RISC-Malawi) from Malawi. PLoS One. 2016 Dec 28;11(12):e0168126. 10.1371/journal.pone.0168126 28030608 PMC5193399

[15] Chew R, Zhang M, Chandna A, Lubell Y. The impact of pulse oximetry on diagnosis, management and outcomes of acute febrile illness in low-income and middle-income countries: a systematic review. BMJ Glob Health. 2021 Nov;6(11):e007282. 10.1136/bmjgh-2021-007282 34824136 PMC8627405

[16] Osier FH, Berkley JA, Ross A, Sanderson F, Mohammed S, Newton CR. Abnormal blood glucose concentrations on admission to a rural Kenyan district hospital: prevalence and outcome. Arch Dis Child. 2003 Jul;88(7):621–5. 10.1136/adc.88.7.621 12818911 PMC1763181

[17] Uleanya ND, Aniwada EC, Nwokoye IC, Ndu IK, Eke CB. Relationship between glycemic levels and treatment outcome among critically ill children admitted into emergency room in Enugu. BMC Pediatr. 2017 May 16;17(1):126. 10.1186/s12887-017-0879-8 28511644 PMC5434620

[18] Baker T, Ngwalangwa F, Masanjala H, Dube Q, Langton J, Marrone G, et al. Effect on mortality of increasing the cutoff blood glucose concentration for initiating hypoglycaemia treatment in severely sick children aged 1 month to 5 years in Malawi (SugarFACT): a pragmatic, randomised controlled trial. Lancet Glob Health. 2020 Dec;8(12):e1546–54. 10.1016/S2214-109X(20)30388-0 33038950

[19] Malawi Demographic and Health Survey 2015–16. Zomba & Rockville: National Statistical Office of Malawi & ICF; 2017. Available from: https://dhsprogram.com/pubs/pdf/FR319/FR319.pdf [cited 2021 Oct 9].

[20] Verbal autopsy standards: the 2016 WHO verbal autopsy instrument. Geneva: World Health Organization; 2016. Available from: https://www.who.int/publications/m/item/verbal-autopsy-standards-the-2016-who-verbal-autopsy-instrument [cited 2020 Feb 20].

[21] Sjoding MW, Luo K, Miller MA, Iwashyna TJ. When do confounding by indication and inadequate risk adjustment bias critical care studies? A simulation study. Crit Care. 2015 Apr 30;19(1):195. 10.1186/s13054-015-0923-8 25925165 PMC4432515

[22] Okoli GN, Sanders RD, Myles P. Demystifying propensity scores. Br J Anaesth. 2014 Jan;112(1):13–5. 10.1093/bja/aet290 24318697 PMC3854550

[23] Becker SO, Ichino A. Estimation of average treatment effects based on propensity scores. Stata J. 2002;2(4):358–77. 10.1177/1536867X0200200403

[24] King C, Zadutsa B, Banda L, Phiri E, McCollum ED, Langton J, et al. Supplementary Appendix file for the manuscript Progression of hypoxaemia and hypoglycaemia in children referred from primary care facilities in Malawi – a prospective cohort study [data repository]. London: Figshare; 2022. 10.6084/m9.figshare.19122032.v1 10.6084/m9.figshare.19122032.v1

[25] IMAI district clinician manual: hospital care for adolescents and adults: guidelines for the management of illnesses with limited resources. Geneva: World Health Organization; 2011. Available from: https://www.who.int/publications/i/item/9789241548281 [cited 2022 Feb 24].

[26] Lungu EA, Darker C, Biesma R. Determinants of healthcare seeking for childhood illnesses among caregivers of under-five children in urban slums in Malawi: a population-based cross-sectional study. BMC Pediatr. 2020 Jan 17;20(1):20. 10.1186/s12887-020-1913-9 31952484 PMC6966883

[27] King C, Dube A, Zadutsa B, Banda L, Langton J, Desmond N, et al. Paediatric Emergency Triage, Assessment and Treatment (ETAT) – preparedness for implementation at primary care facilities in Malawi. Glob Health Action. 2021 Jan 1;14(1):1989807. 10.1080/16549716.2021.1989807 34779363 PMC8592602

[28] Oxner A, Vellanki M, Myers A, Bangura F, Bangura S, Koroma AM, et al. Reducing mortality from severe malaria in Sierra Leonean children by applying the World Health Organization’s standard malarial protocol with additional sublingual glucose: a continuous quality improvement report. Int J Infect Dis. 2020 Jul;96:61–7. 10.1016/j.ijid.2020.04.046 32339722

[29] Graham H, Bakare AA, Ayede AI, Oyewole OB, Gray A, Neal E, et al. Diagnosis of pneumonia and malaria in Nigerian hospitals: a prospective cohort study. Pediatr Pulmonol. 2020 Jun;55(S1) Suppl 1:S37–50. 10.1002/ppul.24691 32074408 PMC7318580

[30] Kilov K, Hildenwall H, Dube A, Zadutsa B, Banda L, Langton J, et al. Integrated Management of Childhood Illnesses (IMCI): a mixed-methods study on implementation, knowledge and resource availability in Malawi. BMJ Paediatr Open. 2021 Apr 30;5(1):e001044. 10.1136/bmjpo-2021-001044 34013071 PMC8098945

[31] Hooli S, King C, Zadutsa B, Nambiar B, Makwenda C, Masache G, et al. The epidemiology of hypoxemic pneumonia among young infants in Malawi. Am J Trop Med Hyg. 2020 Mar;102(3):676–83. 10.4269/ajtmh.19-0516 31971153 PMC7056410

[32] Thangaratinam S, Brown K, Zamora J, Khan KS, Ewer AK. Pulse oximetry screening for critical congenital heart defects in asymptomatic newborn babies: a systematic review and meta-analysis. Lancet. 2012 Jun 30;379(9835):2459–64. 10.1016/S0140-6736(12)60107-X 22554860

[33] Bjornstad E, Preidis GA, Lufesi N, Olson D, Kamthunzi P, Hosseinipour MC, et al. Determining the quality of IMCI pneumonia care in Malawian children. Paediatr Int Child Health. 2014 Feb;34(1):29–36. 10.1179/2046905513Y.0000000070 24091151 PMC4424282

[34] Uwemedimo OT, Lewis TP, Essien EA, Chan GJ, Nsona H, Kruk ME, et al. Distribution and determinants of pneumonia diagnosis using Integrated Management of Childhood Illness guidelines: a nationally representative study in Malawi. BMJ Glob Health. 2018 Apr 9;3(2):e000506. 10.1136/bmjgh-2017-000506 29662688 PMC5898357

[35] Kobayashi M, Mwandama D, Nsona H, Namuyinga RJ, Shah MP, Bauleni A, et al. Quality of case management for pneumonia and diarrhea among children seen at health facilities in southern Malawi. Am J Trop Med Hyg. 2017 May;96(5):1107–16. 10.4269/ajtmh.16-0945 28500813 PMC5417203

[36] Johansson EW, Nsona H, Carvajal-Aguirre L, Amouzou A, Hildenwall H. Determinants of Integrated Management of Childhood Illness (IMCI) non-severe pneumonia classification and care in Malawi health facilities: analysis of a national facility census. J Glob Health. 2017 Dec;7(2):020408–020408. 10.7189/jogh.07.020408 29163934 PMC5680530

[37] Desmond NA, Nyirenda D, Dube Q, Mallewa M, Molyneux E, Lalloo DG, et al. Recognising and treatment seeking for acute bacterial meningitis in adults and children in resource-poor settings: a qualitative study. PLoS One. 2013 Jul 4;8(7):e68163. 10.1371/journal.pone.0068163 23861864 PMC3701660

[38] Chandna A, Osborn J, Bassat Q, Bell D, Burza S, D’Acremont V, et al. Anticipating the future: prognostic tools as a complementary strategy to improve care for patients with febrile illnesses in resource-limited settings. BMJ Glob Health. 2021 Jul;6(7):e006057. 10.1136/bmjgh-2021-006057 34330761 PMC8327814

[39] Resolution A/RES/70/1. Transforming our world: the 2030 agenda for sustainable development. In: Seventieth United Nations General Assembly, New York, 25 September 2015. New York: United Nations; 2015. Available from: http://www.un.org/ga/search/view_doc.asp?symbol=A/RES/70/1&Lang=E [cited 2022 Feb 24].

